# Pharmacological blockade of either cannabinoid CB_1_ or CB_2_ receptors prevents both cocaine-induced conditioned locomotion and cocaine-induced reduction of cell proliferation in the hippocampus of adult male rat

**DOI:** 10.3389/fnint.2013.00106

**Published:** 2014-01-08

**Authors:** Eduardo Blanco-Calvo, Patricia Rivera, Sergio Arrabal, Antonio Vargas, Francisco Javier Pavón, Antonia Serrano, Estela Castilla-Ortega, Pablo Galeano, Leticia Rubio, Juan Suárez, Fernando Rodriguez de Fonseca

**Affiliations:** ^1^Departament de Pedagogia i Psicologia, Facultat de Ciències de l’Educació, Universitat de LleidaLleida, Spain; ^2^Laboratorio de Investigación-UGC de Salud Mental, Instituto de Investigación Biomédica de Málaga, Universidad de Málaga, Hospital Regional Universitario de MálagaMálaga, Spain; ^3^Instituto de Investigaciones Cardiológicas Prof. Dr. Alberto C. Taquini, Universidad de Buenos Aires-CONICETCiudad de Buenos Aires, Argentina; ^4^Departamento de Anatomía y Medicina Legal y Forense, Facultad de Medicina, Universidad de MálagaMálaga, Spain

**Keywords:** cocaine, neurogenesis, cannabinoid receptors, Rimonabant, AM630, inflammation, hippocampus, striatum

## Abstract

Addiction to major drugs of abuse, such as cocaine, has recently been linked to alterations in adult neurogenesis in the hippocampus. The endogenous cannabinoid system modulates this proliferative response as demonstrated by the finding that pharmacological activation/blockade of cannabinoid CB_1_ and CB_2_ receptors not only modulates neurogenesis but also modulates cell death in the brain. In the present study, we evaluated whether the endogenous cannabinoid system affects cocaine-induced alterations in cell proliferation. To this end, we examined whether pharmacological blockade of either CB_1_ (Rimonabant, 3 mg/kg) or CB_2_ receptors (AM630, 3 mg/kg) would affect cell proliferation [the cells were labeled with 5-bromo-2′-deoxyuridine (BrdU)] in the subventricular zone (SVZ) of the lateral ventricle and the dentate subgranular zone (SGZ). Additionally, we measured cell apoptosis (as monitored by the expression of cleaved caspase-3) and glial activation [by analyzing the expression of glial fibrillary acidic protein (GFAP) and Iba-1] in the striatum and hippocampus during acute and repeated (4 days) cocaine administration (20 mg/kg). The results showed that acute cocaine exposure decreased the number of BrdU-immunoreactive (ir) cells in the SVZ and SGZ. In contrast, repeated cocaine exposure reduced the number of BrdU-ir cells only in the SVZ. Both acute and repeated cocaine exposure increased the number of cleaved caspase-3-, GFAP- and Iba1-ir cells in the hippocampus, and this effect was counteracted by AM630 or Rimonabant, which increased the number of BrdU-, GFAP-, and Iba1-ir cells in the hippocampus. These results indicate that the changes in neurogenic, apoptotic and gliotic processes that were produced by repeated cocaine administration were normalized by pharmacological blockade of CB_1_ and CB_2_. The restorative effects of cannabinoid receptor blockade on hippocampal cell proliferation were associated with the prevention of the induction of conditioned locomotion but not with the prevention of cocaine-induced sensitization.

## INTRODUCTION

Addiction is a chronic disorder in which contextual memories play important roles in drug-taking and drug-seeking (Koob and Volkow, 2010). These contextual memories associate specific environments/stimuli with the reinforcing properties of drugs. The hippocampus is likely involved in these responses because of its role in forming context-specific memories that are associated with conditioned responses that boost drug-seeking (McFarland and Kalivas, 2001; Koob and Volkow, 2010). In the case of psychostimulant exposure, repeated administration of either cocaine or amphetamine results in enhanced psychomotor responses that are a form of implicit memory called sensitization. Sensitization is not linked to contextual associations; rather, sensitization is linked to plasticity in the dopaminergic signaling in the circuits of the ventral tegmental area, nucleus accumbens, and dorsal striatum (Pierce and Kalivas, 1997; Carlezon and Nestler, 2002). Understanding how these memories (either contextual or implicit) lead to addicted phenotypes might help to address the problem of preventing the reinstatement of drug-seeking, which is a core problem for addiction therapeutics.

One of the neuroplasticity mechanisms by which habit-forming drugs may lead to addiction is the substitution and incorporation of new neurons and glial cells into reward and memory circuits. The brain retains the ability to continuously generate new cells from progenitors that are located in two main proliferative areas, the subventricular zone (SVZ) of the lateral ventricle and the subgranular zone (SGZ) in the dentate gyrus (DG) of the hippocampus (Gould et al., 1999; Shors et al., 2001). Additional proliferative responses that are associated with the generation of glial cells have been described in the cortex, particularly in the medial prefrontal cortex (Mandyam and Koob, 2012). While the cells generated in the SVZ primarily migrate to the olfactory bulb, some of the migrating cells progress to adjacent areas that include the striatal complex. SGZ cells differentiate, remain in the hippocampal formation, and are thought to be essential for the remodeling of memories associated with biographic events (Shors et al., 2001).

In recent years, compelling evidence has demonstrated that alterations in the rate of cell proliferation in the adult hippocampus may regulate drug-seeking and drug-taking. This phenomenon has been demonstrated for cocaine (Dominguez-Escriba et al., 2006; Noonan et al., 2010), amphetamines (Mandyam et al., 2008; Recinto et al., 2012), alcohol (Herrera et al., 2003; He et al., 2005), nicotine (Shingo and Kito, 2005), cannabinoids (Rueda et al., 2002; Jiang et al., 2005), and opiates (Eisch et al., 2000). Although in some cases (mostly depending on the dose delivered), drugs of abuse enhance neurogenesis [see for instance (Jiang et al., 2005)], these studies have shown that drugs of abuse decrease adult cell proliferation/neurogenesis. The resulting reduction in neuronal turnover sustains drug-seeking by preserving contextual memories associated with drug reward (Mandyam and Koob, 2012). In the case of cocaine, neurogenesis has been linked to the protection of cocaine-primed relapse (Deschaux et al., 2012), and to the facilitation of the extinction of cocaine-induced place preference (Mustroph et al., 2011), whereas the impairment of neurogenesis increases behavioral sensitization in a rat model (Garcia-Fuster et al., 2010), facilitates cocaine self-administration by extending the time required for the extinction of this behavior (Noonan et al., 2010) and generates deficits in working memory (Sudai et al., 2011). Moreover, some of these effects are reversed after cocaine withdrawal (Noonan et al., 2008).

One molecular mechanism that controls cell proliferation in the adult brain, particularly in the hippocampus, is mediated by the endogenous cannabinoid system. This lipid signaling system not only regulates embryonic cell fate in the developing brain but also regulates adult neurogenesis (Aguado et al., 2005; Goncalves et al., 2008; Diaz-Alonso et al., 2012). This effect is mediated through the activations of CB_1_ and CB_2_ receptors (Jin et al., 2004; Hill et al., 2010). The effects of the endogenous cannabinoid system can be bidirectional; i.e., the endogenous cannabinoid system can promote cell proliferation during development or after brain insult (Aguado et al., 2005, 2007) or reduce cell proliferation when overstimulated by the administration of cannabinoids (Rueda et al., 2002; Hill et al., 2006). The contribution of the endogenous cannabinoid system to cocaine-induced alterations in neurogenesis is not known. However, there is clear evidence that both CB_1_ and CB_2_ receptors are linked to the generation of contextual memories associated with cocaine-induced sensitization and cocaine seeking and relapse (Gorriti et al., 1999; De Vries et al., 2001; Gerdeman et al., 2008; Alvaro-Bartolome and Garcia-Sevilla, 2013). To further explore the role of the endogenous cannabinoid system in regulating memories associated with cocaine exposure, the present study examined whether the blockade of either CB_1_ or CB_2_ cannabinoid receptors during repeated administration of cocaine is capable of (a) affecting the acquisition of cocaine-induced sensitization, (b) disrupting the appearance of cocaine-induced conditioned locomotion, or (c) modulating cocaine-induced cell proliferation.

## MATERIALS AND METHODS

### ANIMALS

All experimental animal procedures were performed in compliance with the European Directive 2010/63/EU on the protection of animals used for scientific purposes and with Spanish regulations (RD 53/2013 and 178/2004). Male Wistar rats (approximately 250 g, 10–12 weeks old; Charles Rivers, Barcelona, Spain) were housed in groups of two in cages maintained in standard conditions (Servicio de Estabulario, Facultad de Medicina, Universidad de Málaga) at room temperature (20°± 2°C), 40 ± 5% relative humidity and a 12-h light/dark cycle with a dawn/dusk effect.

### DRUG ADMINISTRATION

Cocaine hydrochloride was obtained from the Alcaliber Company (Toledo, Spain), subsequent to receiving authorization from the Spanish Agency of Medicines and Medical Devices and dissolved in a sterile 0.9% NaCl solution immediately prior to experimentation. Rats were given acute (20 mg/kg) injections, repeated injections (20 mg/kg) and/or an acute challenge injection (priming, 10 mg/kg) of cocaine. Both Rimonabant and AM630 were dissolved in 1% Tween 80 and a sterile 0.9% NaCl solution and administered at a dose of 3 mg/kg. The drugs were injected intraperitoneally (i.p.) in final volumes of 1 ml/kg.

### BrdU ADMINISTRATION

Doses of 50 mg/kg of 5-bromo-2′-deoxyuridine (BrdU, cat. no. B5002, Sigma, St. Louis, MO, USA) were dissolved at 15 mg/mL in a 0.9% saline solution and administered i.p. three times prior to, immediately after and 8 h after the single (acute group) or the final (repeated cocaine injection groups) cocaine doses.

### BEHAVIORAL STUDIES

#### Open field test

Four open field (OF) mazes with gray backgrounds and dimensions (in cm) of L50 × W50 × H50 were used (Panlab, Barcelona, Spain). The arenas were uniformly illuminated by a fluorescent lamp, and the light intensities at the centers of the arenas were 50–70 lx. All behavioral procedures were carried out between 8:00 a.m. and 6:00 p.m. The animals were placed individually in the center of each arena after treatments, and their behaviors were recorded and analyzed for 20 min with a video tracking system (Smart®, Panlab, Barcelona, Spain). The apparatus was cleaned between sessions with 70% ethanol and then dried. Locomotor activity was assessed by measuring the total distance traveled (in cm) by each rat. The testing orders of the groups and the treatments were counterbalanced in all behavioral experiments to avoid confounding effects of the time of the day at which the animals were tested.

#### Handling and acclimation

The animals were weighed, handled (10 min) and habituated daily to the injection procedures (i.e., they were held and pseudo-injected) over 1 week to minimize stress effects. For the pseudo-injection, the lower abdomen of the rat was pressed with a capped syringe. Before beginning the experiment each day, the animals were acclimated to the testing room for 20 min.

#### Locomotor activity after acute and repeated administration of cocaine and co-administration of cocaine and rimonabant or AM630

**FIGURE 1:**
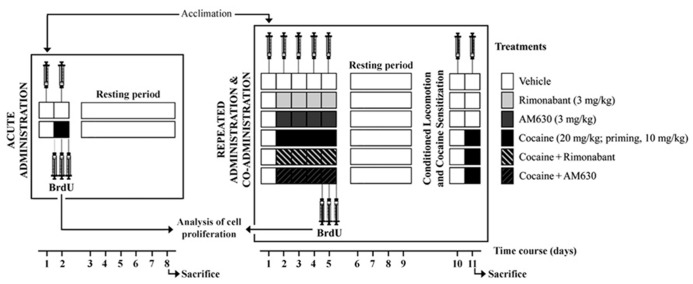
**Schematic representation of the treatments and the behavioral protocols used in this study.** The locomotor activities of two independent groups of rats were evaluated in the OF after acute and repeated administration of cocaine and co-administration of cocaine (20 mg/kg) and Rimonabant (3 mg/kg) or AM630 (3 mg/kg). Conditioned locomotion responses (5 days after last cocaine injection) and cocaine-induced sensitization with priming doses of cocaine (10 mg/kg) were evaluated. After treatments, all animals were tested daily in the OF, and locomotion was measured for 20 min. Three doses of BrdU were administered to each animal prior to, immediately after and 8 h after the last cocaine injection for the analyses of brain cell proliferation.

One group of animals received a vehicle injection, and these animals were immediately placed in the OF for 20 min to measure spontaneous locomotor activity. Twenty-four hours later, the animals were re-exposed to the OF for 20 min to assess the effects of the administration of vehicle or the acute administration of cocaine (20 mg/kg). The animals also received BrdU as described above (**Figure 1**). This group of animals (i.e., the vehicle-injected and cocaine-exposed group) was sacrificed and perfused 6 days after cocaine injection. A separate group of animals was used to assess locomotion responses after co-administration of cocaine with either vehicle, Rimonabant or AM630. The experimental groups that were monitored after acute co-administration of treatments were as follows (*n* = 8/group): vehicle, Rimonabant (3 mg/kg), AM630 (3 mg/kg), cocaine (20 mg/kg), Rimonabant (3 mg/kg) + cocaine (20 mg/kg), and AM630 (3 mg/kg) + cocaine (20 mg/kg). An independent group of animals of the same size and treatment distribution was also tested in the OF for 20 min after the injection of vehicle injection (day 1); over the following 4 days (days 2–5), these animals received repeated administrations of vehicle, Rimonabant, AM630, or cocaine or repeated co-administration of cocaine and Rimonabant or AM630 as described above (*n* = 8/group; **Figure 1**). On the final day of cocaine administration, BrdU was also administered.

#### Cocaine-induced conditioned locomotion and sensitization

After the repeated treatments were completed, the animals were left undisturbed in their home cages from day 5 to day 9 (resting period). On the 10th day, we assessed conditioned locomotion responses and the locomotor activity responses induced by the contextual memories formed through the association of the repeated administrations of cocaine and the place in which the cocaine exerted its stimulant effects. To this end, the animals were injected with vehicle and placed in the OF, and their locomotions were recorded for 10 min. On the following day (day 11), we tested the rats’ responses to a single dose of cocaine (a lower priming dose of cocaine, 10 mg/kg) in the OF context that had previously been associated with cocaine administration (**Figure 1**). The cocaine-induced sensitization responses of the animals were evaluated for 30 min in the OF test.

### SAMPLE COLLECTION

The following seven experimental groups (*n* = 8/group) were processed for histology: vehicle, acute cocaine, repeated cocaine, Rimonabant, AM630, repeated cocaine + Rimonabant and repeated cocaine + AM630. The animals were anesthetized (sodium pentobarbital, 50 mg/kg, i.p.) 1 h after the final administration of vehicle or cocaine in a room that as isolated from the other experimental animals. The animals were then transcardially perfused with 4% paraformaldehyde in 0.1 M phosphate-buffered saline (PBS), and their brains were dissected and maintained in the same fixative solution overnight at 4°C. The brains were then cut into 40-μm-thick coronal sections and divided into five parallel series using a vibratome (Leica VT1000S). The sections were stored at 4°C in PB with 0.002% (w/v) sodium azide until further use for immunohistochemistry.

### BrdU IMMUNOHISTOCHEMISTRY

To analyze cell proliferation in the SVZ of the lateral ventricles and the SGZ of DG, free-floating coronal sections from 2.28 to -0.24 and from -2.16 to -4.20 relative to bregma (Paxinos and Watson, 2007) were selected from one of the five parallel series obtained from each brain of the rats in the seven experimental groups. The sections were first washed several times with PBS to remove the sodium azide and then incubated in a solution of 3% hydrogen peroxide and 10% methanol in 0.1 M PB for 45 min at room temperature in darkness to inactivate the endogenous peroxidase. After three washes in PBS for 10 min, the DNA was denatured by exposing the sections to 2 M HCl for 15 min at 37°C, and this process was followed by two washes in 0.15 M borate buffer to neutralize the pH. After three 10 min washes in PBS, the sections were incubated in a blocking solution containing 0.3% albumin, 0.3% triton X-100, and 0.05% sodium azide in PBS for 45 min. The sections were then incubated overnight in mouse anti-BrdU (1:2000, Hybridoma Bank, Iowa City, IA, USA; ref. G3G4) at 4°C (Cifuentes et al., 2011; Rivera et al., 2011). The following day, the sections were washed three times with PBS, incubated in biotinylated goat anti-mouse immunoglobulin G (IgG; H + L; 1:1000, Pierce, Rockford, IL, USA; cat. no. 31800) for 90 min, washed again in PBS and incubated in avidin–biotin peroxidase complex (Pierce) diluted 1:250 in PBS in darkness at room temperature for 45 min. After three washes in PBS in darkness, immunolabeling was revealed with 0.05% diaminobenzidine (DAB; Sigma), 0.05% nickel ammonium sulfate and 0.03% H_2_O_2_ in PBS. After several washes in PBS, the sections were mounted in slides treated with poly-l-lysine solution (Sigma), air-dried, dehydrated in ethanol, cleared with xylene and coverslipped with Eukitt mounting medium (Kindler GmBH & Co, Freiburg, Germany). Digital high-resolution photomicrographs of the rat brains were taken under constant light, brightness and contrast conditions with an Olympus BX41 microscope equipped with an Olympus DP70 digital camera (Olympus Europa GmbH, Hamburg, Germany). Representative digital images were mounted and labeled using Microsoft PowerPoint 2007.

### CLEAVED Caspase-3, GFAP, AND Iba-1 IMMUNOHISTOCHEMISTRY

To analyze the immunohistochemical expression of cleaved caspase-3, glial fibrillary acidic protein (GFAP), and Iba-1 in the striatum and the hippocampus, free-floating coronal sections from 2.28 to -0.24 and from -2.16 to -4.20 relative to bregma levels (Paxinos and Watson, 2007) were selected from one of five parallel series obtained from each brain of the rats in the seven experimental groups. One immunostaining batch containing 56 slides (seven groups, eight animals each) was stained simultaneously to avoid variations in staining intensity due to the staining procedures. Free-floating sections were first washed several times with PBS to remove the sodium azide and then incubated in sodium citrate (50 mM in dH2O, pH 6) for 30 min at 80°C. This process was followed by three 10 min washes in PBS and incubation in 3% hydrogen peroxide in PBS for 20 min in darkness at room temperature to inactivate endogenous peroxidase. The sections were then incubated in a blocking solution containing 10% serum (donkey or goat), 0.3% triton X-100, and 0.1% sodium azide in PBS for 1 h. The sections were incubated overnight in the following diluted primary antibodies at 4°C: rabbit anti-cleaved caspase-3 (1:500, Cell Signaling; cat. no. 9661), mouse anti-GFAP (1:500, Sigma; cat. no. G3893), or rabbit anti-Iba-1 (1:1000, Wako, Osaka, Japan; cat. no. 019-19741). The following day, the sections were incubated with one of the following secondary antibodies for 1 h as appropriate: biotinylated goat anti-mouse IgG (1:500, Sigma; cat. no. B7264) or biotinylated donkey anti-rabbit IgG (1:500, Amersham, Little Chalfont, England; cat. no. RPN 1004). The sections were then washed three times with PBS and incubated in ExtrAvidin peroxidase (Sigma, St. Louis, MO, USA) diluted 1:2000 in darkness at room temperature for 1 h. After three washes in PBS in darkness, we revealed the immunolabeling with 0.05% DAB (Sigma), 0.05% nickel ammonium sulfate, and 0.03% H2O2 in PBS. The next histological steps were performed as described above. Digital high-resolution photomicrographs of the rat brains were taken using conditions and equipment that were identical to those described above.

### QUANTIFICATION OF BrdU, CLEAVED CASPASE-3, GFAP, AND Iba1-IMMUNOREACTIVE CELLS

To quantify cell proliferation in the SVZ and SGZ and apoptosis and gliosis in the striatum and the hippocampus, immunoreactive (-ir) nuclei and cells that came into focus were manually counted using a standard optical microscope with a 40 × objective. BrdU-ir nuclei in the SVZ and SGZ and cleaved caspase-3-, GFAP- and Iba1-ir cells of the striatum and hippocampal DG, CA3, and CA1 regions were counted from 2.28 to -0.24 and from -2.16 to -4.20 relative to bregma, respectively (Paxinos and Watson, 2007); thus, twenty 40-μm thick coronal sections per animal were used. We considered the same numbers of sections per brain region for each analyzed animal. Cell counts were performed in both cerebral hemispheres in all cases. The results are expressed as the average numbers of ir nuclei/cells per section or area (mm^2^) for each of the following experimental group (*n* = 8/group): vehicle, acute cocaine, repeated cocaine, Rimonabant, AM630, repeated cocaine + Rimonabant and repeated cocaine + AM630.

### QUANTIFICATION OF GFAP IMMUNOREACTIVITY

Digital high-resolution microphotographs of the striatum and hippocampus were taken with a 10 × objective under constant light, brightness and contrast conditions with an Olympus BX41 microscope equipped with an Olympus DP70 digital camera. Densitometric quantifications of the immunoreactivities of representative areas were determined using the ImageJ 1.38X (NIH, MD, USA) analysis software. In each tissue section, we focused on the dorsal striatum, the hippocampal CA1 and CA3 areas of Ammon’s horn and the DG. For both CA areas, we considered the following layers: stratum oriens (SO), stratum pyramidale (SP), stratum radiatum (SR), stratum lucidum (SL), and stratum lacunosum-moleculare (SL-M). For the DG, we considered following layers; the molecular layer (ml), the granular cell layer (gcl) and the polymorphic cell layer (pcl).

### STATISTICAL ANALYSIS

The data are represented as the mean ± the SEM from at least eight animals. Kolmogorov–Smirnov normality tests indicated that all data followed Gaussian distributions (*P* > 0.1); thus, parametric statistical tests were selected. Statistical analyses of the histological data were performed with one-way ANOVA. Behavioral data were analyzed with one- or two-way repeated measures ANOVA with the factors of time (days) and treatment (vehicle, Rimonabant, AM630, cocaine, Rimonabant + cocaine and AM630 + cocaine) followed by Bonferroni *post hoc* tests for multiple comparisons. *P* values less than 0.05 were considered statistically significant.

## RESULTS

### EFFECT OF ACUTE AND REPEATED ADMINISTRATION OF COCAINE AND CO-ADMINISTRATION OF COCAINE AND RIMONABANT OR AM630

**FIGURE 2:**
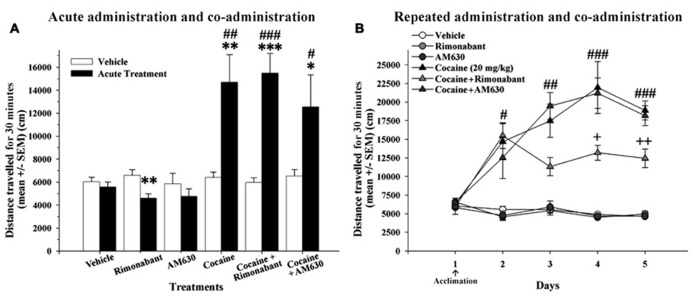
**(A)** Effects of acute administration of cocaine and co-administration of cocaine (20 mg/kg) and Rimonabant (3 mg/kg) or AM630 (3 mg/kg) in the OF test. The acute cocaine-treated groups (cocaine, cocaine + Rimonabant and cocaine + AM630) exhibited significant increases in locomotor activity compared to the previous vehicle administration on day 1 (^*^P < 0.05, ^**^P < 0.01, ^***^P < 0.001) and compared to the vehicle group (^#^*P* < 0.05, ^##^*P* < 0.01, ^###^*P* < 0.001). **(B)** The effects of repeated administration of cocaine and co-administration of cocaine (20 mg/kg) and Rimonabant (3 mg/kg) or AM630 (3 mg/kg) in the OF test. The repeated cocaine-treated groups (cocaine, cocaine + Rimonabant and cocaine + AM630) exhibited greater locomotion than did the groups that did not receive cocaine (vehicle, Rimonabant and AM630) on days 2 (^#^*P* < 0.05), 3 (^#^^#^*P* < 0.01), 4 and 5 (^###^*P* < 0.001). The cocaine + Rimonabant group exhibited reduced horizontal locomotion on days 4 and 5 compared to the other repeated cocaine-treated groups (cocaine and cocaine + AM630; ^+^*P* < 0.05, ^+^^+^*P* < 0.01). All results are presented as the means ± the SEMs (*n* = 8/group).

To study the interaction of the pharmacological blockade of CB_1_ and CB_2_ receptors and the acute stimulating effects of cocaine, we evaluated locomotor responses to acute doses of cannabinoid receptor antagonists (Rimonabant or AM630 at 3 mg/kg, i.p.) that were administered alone or in combination with cocaine (20 mg/kg, i.p.; **Figure 2A**). All animals received an administration of vehicle on day 1 and the treatment administration 24 h later. A two-way ANOVA revealed that the main effects of group and day and the interaction between group and day were all significant [*F*(5,42) = 8.39, *P* < 0.001; *F*(1,42) = 21.71, *P* < 0.001; *F*(5,42) = 8.36, *P* < 0.001, respectively]. *Post hoc* comparisons revealed that the groups that received acute injections cocaine (alone and in combination with Rimonabant or AM630) exhibited similar significant increases in locomotion (**Figure 2A**). Moreover, *post hoc* analysis indicated that these groups displayed significantly enhanced locomotor activity compared to the groups that received an acute dose of vehicle or CB_1_ and CB_2_ antagonists during the second exposure to the OF (**Figure 2A**). Further *post hoc* comparisons revealed a reduction in locomotion after a single administration of Rimonabant. In general, these results indicate that acute co-administration of CB_1_ and CB_2_ antagonists with cocaine did not affect the acute stimulating effects of cocaine.

To determine whether cannabinoid receptor antagonism modulates the acquisition of cocaine conditioning processes in the OF, we assessed the effects of repeated co-administration of CB_1_ and CB_2_ antagonists with cocaine. After an initial day on which all experimental groups received vehicle injection, the animals received repeated administrations of vehicle, Rimonabant, AM630, or cocaine or co-administration of cocaine and Rimonabant or AM630 for four consecutive days (**Figure 2B**). A repeated-measures ANOVA revealed that the main effects of treatment [*F*(5,42) = 52.57, *P* < 0.001)], day [*F*(4,168) = 17.82, *P* < 0.001], and the day x treatment interaction [*F*(20,168) = 7.41, *P* < 0.001] were all significant. *Post hoc* comparisons indicated that the three groups that received cocaine (alone or in combination with CB_1_ and CB_2_ antagonists) exhibited significantly increased locomotor activity over all 4 days of cocaine conditioning acquisition relative to the vehicle-, Rimonabant- and AM630-treated groups (no differences between these latter groups were found). However, *post hoc* analyses also revealed that the cocaine + Rimonabant group exhibited reduced horizontal locomotion on days 3 and 4 relative to the other groups that received repeated cocaine injections (i.e., the cocaine and cocaine + AM630 groups; **Figure 2B**). These results may indicate that the co-administration of Rimonabant may attenuate the acquisition of cocaine-sensitized responses.

### EFFECT OF REPEATED ADMINISTRATION OF COCAINE AND CO-ADMINISTRATION OF COCAINE AND RIMONABANT OR AM630 ON CONDITIONED LOCOMOTION AND SENSITIZATION RESPONSES

**FIGURE 3:**
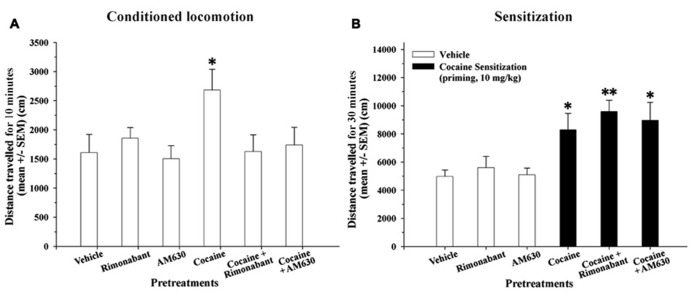
**(A)** Effects of repeated administration of cocaine and co-administration of cocaine (20 mg/kg) and Rimonabant (3 mg/kg) or AM630 (3 mg/kg) on the conditioned locomotion response measured in the OF test. Five days after the final cocaine injection, only the cocaine-treated group exhibited elevated conditioned locomotion responses compared to the vehicle group (^*^P < 0.05) after a vehicle injection. **(B)** Effects of repeated administration of cocaine and co-administration of cocaine (20 mg/kg) and Rimonabant (3 mg/kg) or AM630 (3 mg/kg) on cocaine-induced sensitization measured in the OF test. The repeated cocaine-treated groups (cocaine, cocaine + Rimonabant and cocaine + AM630) exhibited significantly enhanced cocaine-sensitized responses after receiving an acute challenge injection (priming, 10 mg/kg) compared to the groups that did not receive cocaine (vehicle, Rimonabant and AM630; ^*^P < 0.05, ^**^P < 0.01). All results are presented as the means ± the SEMs (*n* = 8/group).

Repeated injections of cocaine-induced a conditioned locomotion response in the cocaine-treated group relative to the vehicle group (*P* < 0.05). Although the cocaine + Rimonabant and cocaine + AM630 groups were repeatedly injected with cocaine, only the cocaine-treated group exhibited a normal conditioned locomotor response (**Figure 3A**). The attenuated expression of conditioned locomotion in the cocaine + Rimonabant group may have been due to weaker acquisition of cocaine conditioning (days 3–4; **Figure 2B**).

On the following day, we measured cocaine-induced sensitization with an acute challenge injection of cocaine (priming, 10 mg/kg). A one-way ANOVA revealed that the main effect of group was significant [*F*(5,45) = 5.17, *P* < 0.001]. Our results revealed that the groups that received repeated cocaine injections (20 mg/kg) alone or in combination with CB_1_ and CB_2_ antagonists (i.e., the cocaine + Rimonabant and cocaine + AM630 groups) exhibited significantly increased locomotor activity compared to their control groups (*P* < 0.05 and 0.01, respectively; **Figure 3B**) as a result of cocaine-induced sensitization. In brief, cannabinoid receptor antagonism (with Rimonabant or AM630) did not affect the expression of cocaine-induced sensitization.

### EFFECT OF ACUTE AND REPEATED ADMINISTRATION OF COCAINE AND CO-ADMINISTRATION OF COCAINE WITH RIMONABANT OR AM630 ON NEWBORN CELLS IN RELEVANT NEUROGENIC ZONES

**FIGURE 4:**
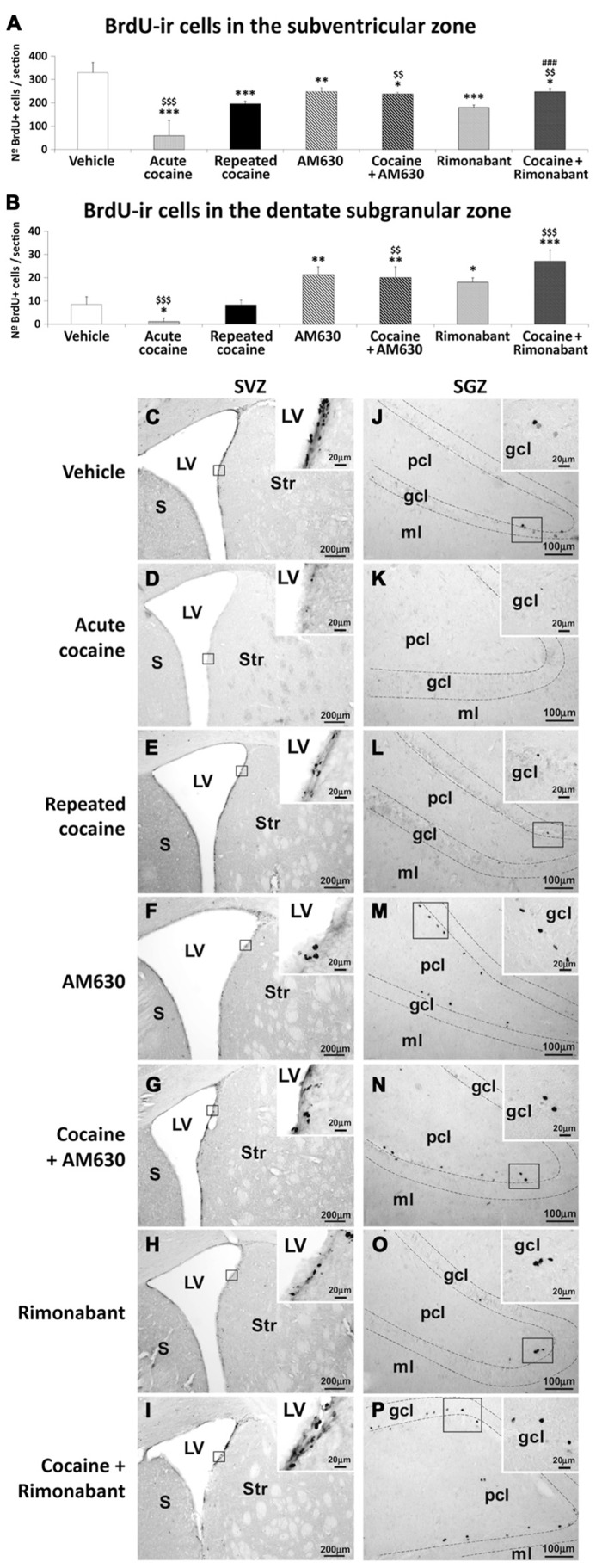
**Effects of repeated administration of cocaine and co-administration of cocaine (20 mg/kg) and Rimonabant (3 mg/kg) or AM630 (3 mg/kg) on cell proliferation in the SVZ (A) and the SGZ (B) as measured with BrdU immunohistochemistry.** Repeated AM630 or Rimonabant administration increased the numbers of BrdU-ir cells observed in the SVZ and SGZ of the repeated cocaine-treated rats. **(C–P)** Representative microphotographs showing low- and high-magnification (insets) views of the typical clusterings of newborn cells in the subventricular zone of the lateral ventricle and the inner border of the granular cell layer. The histogram displays the means ± SEMs (*n* = 8/group) of the numbers of BrdU-ir nuclei per area (mm^2^). One-way ANOVA: ^*^P < 0.05, ^**^P < 0.01, ^***^P < 0.001 vs. vehicle group; ^$$^*P* < 0.01, ^$$$^*P* < 0.001 vs. repeated cocaine group; ^###^*P* < 0.001 vs. Rimonabant group. Scale bars are included in each image.

To investigate the effects of acute and repeated cocaine administration and co-administration of cocaine with the CB_1_ and CB_2_ antagonists Rimonabant and AM630 on cell proliferation in the relevant neurogenic zones, we evaluated newborn cells in the SVZ and SGZ via analyses of BrdU labeling (50 mg/kg; (**Figure 4**). Both the acute and repeated cocaine-treated rats exhibited lower numbers of BrdU-ir cells in the SVZ compared to the vehicle-treated rats (^***^P < 0.001; **Figures 4A,C–E**). Interestingly, the numbers of BrdU-ir cells were significantly lower in the SVZs of the acute compared to the repeated cocaine-treated rats (^$$$^*P* < 0.001). Moreover, both AM630 and Rimonabant also produced significant reductions in the numbers of BrdU-ir cells of the SVZ compared to vehicle (^**^P < 0.01 and ^***^P < 0.001, respectively; **Figures 4A,F,H**). Repeated AM630 or Rimonabant co-administration with cocaine resulted in further significant reductions in the numbers of BrdU-ir cells observed in the SVZs of the repeated cocaine-treated rats (^$$^*P* < 0.01; **Figures 4A,G,I**).

Lower numbers of BrdU-ir cells were observed in the SGZs of the acute cocaine-treated rats than in the vehicle-treated rats (^*^P < 0.05) or the repeated cocaine-treated rats (^$$$^*P* < 0.001; **Figures 4B,J–L**). Thus, no differences in the numbers of BrdU-ir cells were found in the SGZs of the repeated cocaine-treated rats compared to the vehicle rats. In contrast, both AM630 and Rimonabant induced increases in the numbers of BrdU-ir cells in the SGZ compared to vehicle (^**^P < 0.01 and ^*^P < 0.05, respectively; **Figures 4B,M,O**). The increases in the numbers of BrdU-ir cells after repeated AM630 or Rimonabant administration were maintained (^$$^*P* < 0.01) and increased (^$$$^*P* < 0.001), respectively, in the SGZs of repeated cocaine-treated rats (**Figures 4B,N,P**).

### EFFECT OF ACUTE AND REPEATED ADMINISTRATION AND CO-ADMINISTRATION OF COCAINE WITH RIMONABANT OR AM630 ON APOPTOTIC CELLS IN THE STRIATUM AND THE HIPPOCAMPUS

**FIGURE 5:**
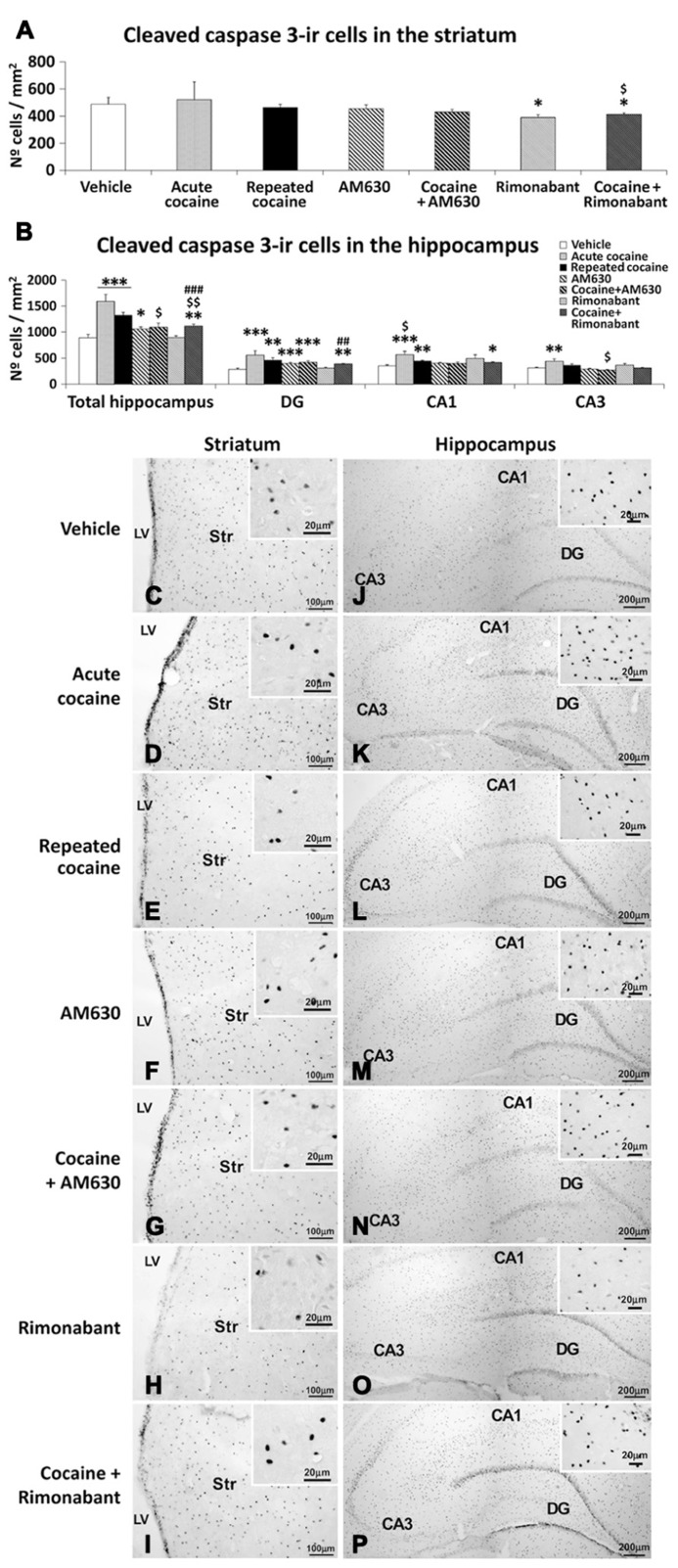
**Effects of repeated administration of cocaine and co-administration of cocaine (20 mg/kg) and Rimonabant (3 mg/kg) or AM630 (3 mg/kg) on cell apoptosis in the striatum (A) and hippocampus (B) as measured with cleaved caspase-3 immunohistochemistry.** Rimonabant administration decreased the numbers of cleaved caspase 3-ir cells observed in the striata and hippocampi of the repeated cocaine-treated rats. Only AM630 decreased the numbers of cleaved caspase 3-ir cells in the hippocampi of the repeated cocaine-treated rats. **(C–P)** Representative microphotographs showing low- and high-magnification (insets) views of apoptotic cells in the striatum and hippocampus. The histogram displays the means ± SEMs (*n* = 8/group) of the numbers of cleaved caspase 3-ir cells per area (mm^2^). One-way ANOVA: ^*^P < 0.05, ^**^P < 0.01, ^***^P < 0.001 vs. vehicle group; ^$^*P* < 0.05, ^$$^*P* < 0.01 vs. repeated cocaine group; ^##^*P* < 0.01, ^###^*P* < 0.001 vs. Rimonabant group. Scale bars are included in each image.

Apoptotic cells, as determined by cleaved caspase-3 immunostaining, were assessed in the striata and the hippocampal DG, CA1, and CA3 areas of rats that were administered acute or repeated cocaine with in combination with the CB_1_ and CB_2_ antagonists Rimonabant and AM630, respectively (**Figure 5**). No differences in the numbers of cleaved caspase 3-ir cells were detected in the striata of the acute or repeated cocaine-treated rats compared to the vehicle-treated rats (**Figures 5A,C–E**). Rimonabant, but not AM630, produced a small but significant reduction in the number of cleaved caspase 3-ir cells in the striatum (^*^P < 0.05; **Figures 5A,F,H**). This reduction in the number of cleaved caspase 3-ir cells in the striatum was also present after the co-administration of cocaine and Rimonabant compared to the vehicle- or repeated cocaine-treated rats (^*^P < 0.05, ^$^*P* < 0.05; **Figures 5A,G,I**).

Increased numbers of cleaved caspase 3-ir cells were observed in the hippocampi of both acute and repeated cocaine-treated rats (^***^P < 0.001; **Figures 5B,J–L**). Regarding the three hippocampal areas analyzed (i.e., the DG, CA1, and CA3), increased numbers of cleaved caspase 3-ir cells were found in the DG and CA1 areas of both the acute and repeated cocaine groups (^***^P < 0.001 and ^**^P < 0.01, respectively) and in the CA3 area of the acute cocaine group (^**^P < 0.01). In contrast, no increase was detected in the CA3 areas of the repeated cocaine-treated rats (**Figures 5B,J–L**). AM630 but not Rimonabant produced a small increase in the number of cleaved caspase 3-ir cells in the hippocampus (^*^P < 0.05), and this result was the a consequence of the prominent increase in the number of cleaved caspase 3-ir cells that was specifically observed in the DG (^***^P < 0.001) and not in the CA1 and CA3 fields (**Figures 5B,M,O**). Interestingly, we observed decreases in the numbers of hippocampal cleaved caspase 3-ir cells after repeated AM630 or Rimonabant administration in the repeated cocaine-treated rats (^$^*P* < 0.05 and ^$$^*P* < 0.01, respectively; **Figures 5B,N,P**).

### EFFECTS OF ACUTE AND REPEATED ADMINISTRATION OF COCAINE AND CO-ADMINISTRATION OF COCAINE WITH RIMONABANT or AM630 ON ASTROGLIOSIS IN THE STRIATUM AND THE HIPPOCAMPUS

**FIGURE 6:**
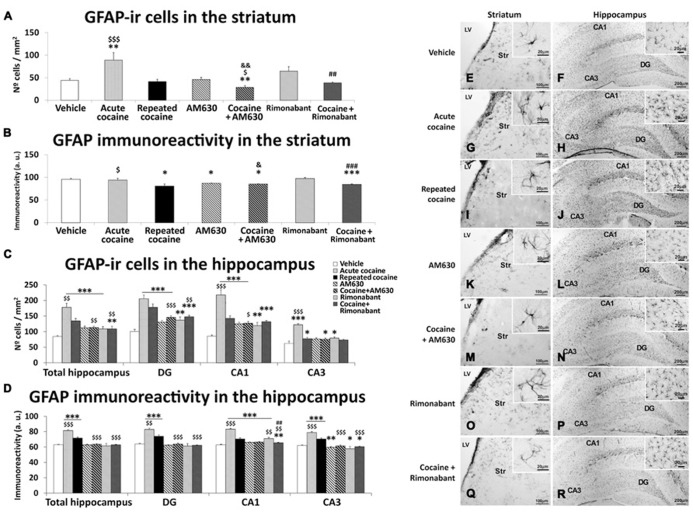
**Effects of repeated administration of cocaine and co-administration of cocaine (20 mg/kg) and Rimonabant (3 mg/kg) or AM630 (3 mg/kg) on astrogliosis in the striatum (A,B) and the hippocampus (C,D) as measured with GFAP immunohistochemistry.** AM630 administration decreased the numbers of GFAP-ir cells observed in the striata of the repeated cocaine-treated rats. Both AM630 and Rimonabant induced decreases in the numbers of GFAP 3-ir cells and reduced the intensities of GFAP immunoreactivity in the hippocampi of the repeated cocaine-treated rats. **(E–R)** Representative microphotographs showing low- and high-magnification (insets) views of astroglial cells in the striatum and hippocampus. The histogram displays the means ± the SEMs (*n* = 8/group) of the numbers of GFAP-ir cells per area (mm^2^) and GFAP immunoreactivities (arbitrary units). One-way ANOVA: ^*^P < 0.05, ^**^P < 0.01, ^***^P < 0.001 vs. vehicle group; ^$^*P* < 0.05, ^$$^*P* < 0.01, ^$$$^*P* < 0.001 vs. repeated cocaine group; ^&^*P* < 0.05, ^&&^*P* < 0.01 vs. AM630 group; ^##^*P* < 0.01, ^###^*P* < 0.001 vs. Rimonabant group. Scale bars are included in each image.

To investigate the effects of acute and repeated cocaine administration and the co-administration of the CB_1_ and CB_2_ antagonists Rimonabant and AM630 on astrogliosis, we evaluated the intensities of GFAP immunoreactivity and the numbers of astrocytes expressing GFAP in the striatum and the hippocampal DG, CA1, and CA3 areas (**Figure 6**). A greater number of GFAP-ir cells were observed in the striatum of the acute cocaine-treated rats compared to the vehicle-treated (^**^P < 0.01) and repeated cocaine-treated rats (^$$$^*P* < 0.001; **Figures 6A,E–G**). Thus, no differences in the numbers of GFAP-ir cells were observed in the striata of the repeated cocaine-treated rats compared to the vehicle-treat rats. Moreover, the striata of the rats treated with AM630 or Rimonabant did not exhibit differences in the numbers of GFAP-ir cells when compared to the vehicle group (**Figures 6A,H,J**). A specific decrease in the numbers of GFAP-ir cells was observed in the striata of the repeated cocaine-treated rats that were co-administered AM630 (^$^*P* < 0.05) but not Rimonabant (**Figures 6A,I,K**). Analyses of GFAP immunoreactivity intensities in the striatum (**Figure 6B**) revealed no differences between the vehicle-, acute cocaine- and repeated Rimonabant groups (**Figures 6B,E,F,J**). In contrast, decreases in GFAP immunostaining were detected in the striata of the repeated cocaine- and AM630-treated rats (^*^P < 0.05; **Figures 6B,G,H**). No effects of repeated AM630 or Rimonabant treatment on GFAP expression were observed in the striata of the repeated cocaine-treated rats (**Figures 6B,I,K**).

Greater numbers of GFAP-ir cells were observed in the hippocampi of the acute and repeated cocaine-treated rats and the AM630 and Rimonabant-treated rats compared to the vehicle-treated rats (^***^P < 0.001; **Figures 6C,L–N,O,Q**). These effects were accompanied by different levels of significance across the three analyzed regions of the hippocampus (i.e., the DG, CA1, and CA3). Notably, the increase in the number of GFAP-ir cells was significantly greater in the hippocampi of the acute cocaine group than in the hippocampi of the remaining groups including the repeated cocaine group (^$$^*P* < 0.01). Specifically, this difference in GFAP-ir cell numbers between the acute and repeated cocaine groups was observed in the CA1 and CA3 fields (^$$$^*P* < 0.001) and not in the DG. The hippocampi of the repeated cocaine-treated rats that also received repeated AM630 or Rimonabant exhibited decreases in the numbers of GFAP-ir cells (^$$^*P* < 0.01), particularly in the DG (^$$$^*P* < 0.001 and ^$$^*P* < 0.01 for the AM630 and Rimonabant co-administration groups, respectively) and not in the CA3 field (**Figures 6C,P,R**). Analyses of GFAP immunoreactivity intensities in the hippocampus (**Figure 6D**) revealed more intense immunostaining in the acute and repeated cocaine-treated rats compared to the vehicle-treated rats (^***^P < 0.001; **Figures 6D,L–N**). Across all hippocampal regions analyzed (i.e., DG, CA1, and CA3), GFAP immunoreactivity was more intense in the acute cocaine-treated rats than in the repeated cocaine-treated rats (^$$^*P* < 0.01). Notably, no effects of pharmacological blockade of CB_1_ or CB_2_ receptors on GFAP immunoreactivities were observed when the total hippocampi were analyzed (**Figures 6D,O,Q**). However, decreases in GFAP immunostaining were specifically observed in the CA3 fields of rats that were administered AM630 (^**^P < 0.01) or Rimonabant (^*^P < 0.05) compared to the vehicle group. Moreover, repeated administrations of AM630 or Rimonabant induced decreases in GFAP immunostaining in the hippocampi of the repeated cocaine-treated rats (**Figures 6D,P,R**).

### EFFECTS OF ACUTE AND REPEATED ADMINISTRATION OF COCAINE AND CO-ADMINISTRATION OF COCAINE WITH RIMONABANT OR AM630 ON MICROGLIOSIS IN THE STRIATUM AND THE HIPPOCAMPUS

**FIGURE 7:**
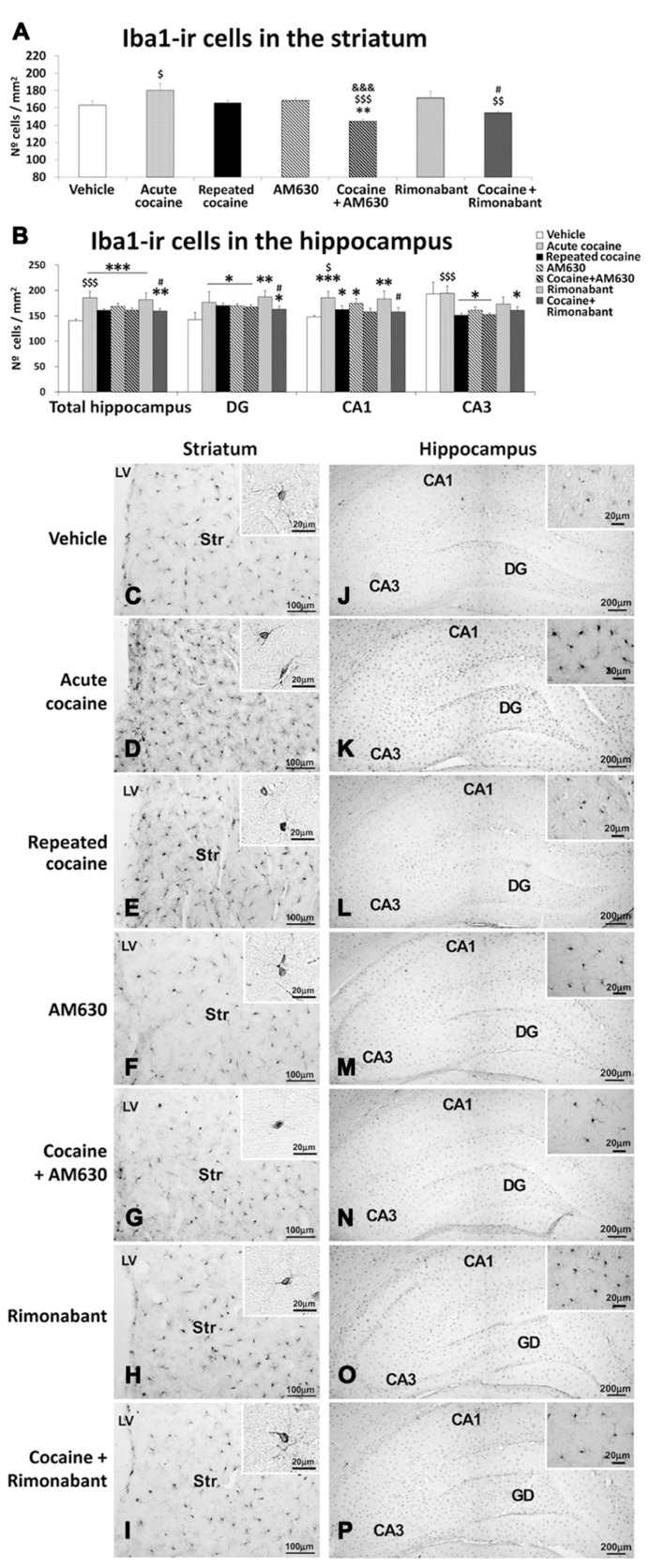
**Effects of repeated administration of cocaine and co-administration of cocaine (20 mg/kg) and Rimonabant (3 mg/kg) or AM630 (3 mg/kg) on microgliosis in the striatum (A)** and the hippocampus **(B)** as measured with Iba-1 immunohistochemistry. AM630 and Rimonabant administration decreased the numbers of Iba1-ir cells observed in the striata but not the hippocampi of the repeated cocaine-treated rats. **(C–P)** Representative microphotographs showing low- and high-magnification (insets) views of microglial cells in the striatum and hippocampus. The histogram displays the means ± the SEMs (*n* = 8/group) of the numbers of Iba1-ir cells per area (mm^2^). One-way ANOVA: ^*^P < 0.05, ^**^P < 0.01, ^***^P < 0.001 vs. vehicle group; ^$^*P* < 0.05, ^$$^*P* < 0.01, ^$$$^*P* < 0.001 vs. repeated cocaine group; ^&&&^*P* < 0.001 vs. AM630 group; ^#^*P* < 0.05 vs. Rimonabant group. Scale bars are included in each image.

To investigate the effects of the acute and repeated cocaine administration and co-administration with the CB_1_ and CB_2_ antagonists Rimonabant and AM630 on microgliosis, we evaluated the numbers of cells that expressed Iba1 in the striatum and the hippocampal DG, CA1, and CA3 areas (**Figure 7**). No differences in the numbers of Iba-ir cells were observed in the striata of the acute or repeated cocaine-treated rats or the AM630- or Rimonabant-treated rats compared to the vehicle-treated rats (**Figures 7A,C–F,H**). However, the numbers of Iba1-ir cells were higher in the striata of the acute cocaine-treated rats than in the repeated cocaine-treated rats (^$^*P* < 0.05; **Figures 7A,C–E**). Interestingly, we observed decreases in the numbers of striatal Iba1-ir cells after repeated AM630 or Rimonabant administration in the repeated cocaine-treated rats (^$$$^*P* < 0.001 and ^$$^*P* < 0.01, respectively; **Figures 7A,G,I**).

Increased numbers of Iba1-ir cells were observed in the hippocampi of both the acute and repeated cocaine-treated rats (^***^P < 0.001; **Figures 7B,J–L**). Regarding the three analyzed hippocampal areas (i.e., the DG, CA1, and CA3), increased numbers of Iba1-ir cells were found in the DG and CA1 areas of both the acute and repeated cocaine groups (^*^P < 0.05). In contrast, no changes and a decrease (^*^P < 0.05) in the number of Iba1-ir cells were detected in the CA3 areas of the acute and repeated cocaine groups, respectively. Notably, the increase in the number of Iba1-ir cells was significantly greater in the hippocampus of the acute cocaine group than in the repeated cocaine group (^$$$^*P* < 0.001). Specifically, this difference in Iba1-ir cell numbers between the acute and repeated cocaine groups was observed in the CA1 and CA3 fields (^$^*P* < 0.05 and ^$$$^*P* < 0.001, respectively) and not in the DG (**Figures 7B,J–L**). Greater numbers of Iba1-ir cells were also observed in the hippocampi of the AM630 and Rimonabant-treated rats compared to the vehicle-treated rats (^***^P < 0.001; **Figures 7B,M,O**). The levels of significance of these effects varied across the three analyzed hippocampal regions (i.e., the DG, CA1, and CA3). Notably, AM630 and Rimonabant failed to affect the numbers of Iba1-ir cells in the hippocampi (or the specific hippocampal regions) in the repeated cocaine-treated rats (**Figures 7B,N,P**).

## DISCUSSION

The present study contributes three main new findings to the understanding of the role of cell proliferation in cocaine addiction. First, pharmacological blockade of either CB_1_ or CB_2_ cannabinoid receptors during the acquisition of behavioral sensitization in a specific context affects hippocampal-dependent conditioned locomotion but not the expression of sensitized responses to cocaine. Second, the absence of cocaine-induced conditioned locomotion in the cannabinoid antagonist-treated animals was associated with enhancements of cell proliferation in the hippocampus. Third, cocaine-induced an activation of the glial compartment that was partially counteracted by the blockade of either CB_1_ or CB_2_ receptors. In the SVZ, repeated cocaine decreased cell proliferation, and the blockade of either of the cannabinoid receptors partially counteracted this effect. Overall, the present study demonstrated that the endogenous cannabinoid system participates in the effects of cocaine on both brain cell proliferation and the emergence of conditioned responses associated with the administration of conditioned responses associated with the administration of cocaine (**Table 1**).

**Table 1:** Summary of the main effects of acute and repeated administration and co-administration of cocaine with Rimonabant or AM630 on factors related to cell proliferation, apoptosis and gliosis.

	Proliferation (BrdU)		Apoptosis (Caspase 3)		Astrogliosis (GFAP)		Microgliosis (Iba1)	
	SVZ	SGZ	Str	HC	Str	HC	Str	HC
Acute cocaine^1^	↓	↓	ns	↑	↑	↑	ns	↑
Repeated cocaine^1^	↓	ns	ns	↑	ns	↑	ns	↑
AM630^1^	↓	↑	ns	↑	ns	↑	ns	↑
Rimonabant^1^	↓	↑	↓	ns	ns	↑	ns	↑
Cocaine + AM630^2^	↑	↑	ns	↓	↓	↓	↓	ns
Cocaine + Rimonabant^2^	↑	↑	↓	↓	ns	↓	↓	ns

1 Effects of acute cocaine, repeated cocaine, AM630 and Rimonabant versus vehicle.

2 Effects of repeated cocaine + AM630 or Rimonabant versus repeated cocaine.

Conditioned locomotion is a conditioned behavioral response in which hippocampus-dependent contextual memories of previous cocaine exposure boost behavioral arousal and locomotion. In a recent study, we demonstrated this link in a mouse model with disruptions of glutamatergic transmission and deficits in neurogenesis in the hippocampus; in the Málaga variant of the lysophosphatidic acid type 1 receptor knockout mice (Matas-Rico et al., 2008; Blanco et al., 2012), hippocampal alterations are associated with profound disruptions of cocaine-induced conditioned locomotion responses (Blanco et al., 2012) but not cocaine-induced sensitization. Although in the present work we do not provide direct evidence linking the cell proliferation responses activated by cannabinoid receptor blockade to the disappearance of conditioned locomotion, there is growing evidence linking the restoration of hippocampal neurogenesis to extinction and protection against cocaine seeking behaviors (Noonan et al., 2008, 2010; Mandyam and Koob, 2012). Our results indicate that the impairments of cell proliferation induced by cocaine are counteracted by pharmacological blockade of either the CB_1_ or CB_2_ cannabinoid receptor, which corroborates the previous suggestion that cell proliferation events in the adult neurogenic niche are under the control of both cannabinoid receptors (Aguado et al., 2005; Diaz-Alonso et al., 2012). In fact, dopamine D2/D3 receptor activation has been linked to both, enhancement of anandamide release and reduction of neurogenesis, suggesting a convergent mechanism for dopamine and anandamide in the inhibition of cell proliferation (Rueda et al., 2002; Deschaux et al., 2012). Although the effects on cell proliferation were more pronounced after cannabinoid CB_1_ receptor blockade, the well-known actions of CB_1_ blockade on anxiety precludes the use of cannabinoid CB_1_ receptor blockers as a restorative therapy in addiction (Navarro et al., 1997). Indeed, cannabinoid CB_2_ receptor-mediated responses are much more interesting, and there is evidence that genetic manipulation or pharmacological blockade of cannabinoid CB_2_ receptors modulates cocaine-addiction related behaviors (Xi et al., 2011; Aracil-Fernandez et al., 2012). Based on the present results, we speculate that the positive outcomes of those studies also relied on the restoration of neurogenesis in the hippocampus. Additionally, increased cell proliferation in the hippocampus due to pharmacological blockade of cannabinoid receptors in the cocaine-treated rats was associated with decreases in mortality (see the cleaved-caspase expression results), which suggests that these new cells had the opportunity to mature and become fully functional. In any case, further studies to address the destinies (in terms of phenotype and circuit integration) and roles of these new hippocampal cells in the contextual memories associated with cocaine administration are underway.

An interesting aspect of the present results is the dissociation between the effects of cannabinoid receptor blockade on conditioned locomotion and sensitization of the responses to cocaine. This dissociation supports the existence of multiple independent circuits that differentially adapt to the effects of cocaine and the differential contributions of endocannabinoids to these circuits. While cannabinoid regulation of the hippocampal memories associated with cocaine has been implicated in the acquisition of cocaine self-administration and cue-induced relapse (De Vries et al., 2001), the actions of cannabinoid receptors on sensitization to psychostimulants are controversial. There are reports that indicate a clear role of cannabinoid receptors in the acquisition of this behavioral adaptation to repeated cocaine exposure (Gerdeman et al., 2008; Thiemann et al., 2008; Mereu et al., 2013). However, there are also reports that indicate that the genetic deletion of cannabinoid receptors has no influence on this effect of cocaine (Lesscher et al., 2005; Thiemann et al., 2008). Under our experimental conditions, we found that blockade of the cannabinoid CB_1_ receptor delayed the acquisition of sensitization, but after the animals were challenged with a 6-day period of incubation, the response to cocaine was equally increased. This finding indicates that, although interference with the molecular mechanisms controlled by the CB_1_ receptors in the basal ganglia may interfere with the molecular adaptations associated with repeated cocaine exposure, the sensitized response is ultimately acquired and expressed. Moreover, the findings that cell proliferation in the adjacent SVZ is increased by either CB_1_ or CB_2_ blockade suggests that this response is apparently not related to the sensitization process, which primarily relies on dopamine-glutamate interactions in dopaminoceptive medium-spiny neurons in the basal ganglia (Carlezon and Nestler, 2002).

Finally, the ability of cannabinoid CB_1_ receptor blockade to attenuate the glial response to cocaine is an interesting finding of this study. As has been previously described in the medial prefrontal cortex, both the astroglial and microglial compartments were activated after cocaine exposure (Mandyam et al., 2007). Pharmacological blockade of either cannabinoid CB_1_ or CB_2_ receptors attenuated this reactivity and normalized the glial compartments of the animals that received cocaine. Both glial cell types are relevant to the induction of plastic changes in the hippocampus and striatum, and the effects of the functions of these cells on cocaine-associated behaviors demand further investigation. However, preliminary studies have clearly indicated that astrocytes, through the regulation of glutamate dynamics, contribute to cocaine addiction (Mandyam et al., 2007; Mandyam and Koob, 2012). In fact, cocaine administration is capable of regulating the expression and activity of two of the main enzymes that regulate synthesis of glutamate and are expressed in astrocytes, the K and L glutaminase isoforms (Blanco et al., 2012). In contrast, the inflammatory pattern of microglia reactivity after cocaine administration suggests that cannabinoid receptors might regulate this response, which is thought to contribute to the plastic changes that result from cocaine exposure.

Overall, the results of the present study suggest that the endogenous cannabinoid system is a key regulatory element of the plastic changes that are associated with cocaine-induced behavioral responses in the rat. This modulatory role is mediated by the signaling processes that are activated by both cannabinoid CB_1_ and CB_2_ receptors. The neuroadaptations to cocaine that are under cannabinoid control include cell proliferation, cell survival and glial activation. Given the clinical withdrawal of cannabinoid CB_1_ antagonists, our results suggest that cannabinoid CB_2_ receptor antagonists are suitable targets for the development of therapies for cocaine addiction.

## AUTHOR CONTRIBUTIONS

Fernando Rodríguez de Fonseca and Juan Suárez conceived and designed the study. Eduardo Blanco-Calvo, Francisco Javier Pavón, and Antonia Serrano performed the behavioral experiments. Patricia Rivera, Sergio Arrabal, Estela Castilla-Ortega, and Pablo Galeano were responsible for the immunohistochemistry analysis. Juan Suárez, Leticia Rubio, and Francisco Javier Pavón performed the statistical analyses and designed the figures. Fernando Rodríguez de Fonseca, Juan Suárez, and Eduardo Blanco-Calvo wrote the manuscript.

## Conflict of Interest Statement

The authors declare that the research was conducted in the absence of any commercial or financial relationships that could be construed as a potential conflict of interest.

## Abbreviations

BrdU: 5-bromo-2′-deoxyuridine
CA1/3: field 1/3 of the Cornu Ammonis
DG: dentate gyrus
gcl: granular cell layer
ml: molecular layer
pcl: polymorphic cell layer
SGZ: subgranular zone of dentate gyrus
SL-M: stratum lacunosum-moleculare
SO: stratum oriens
SP: stratum pyramidale
SR: stratum radiatum
SVZ: subventricular zone of the lateral ventricles.
